# Aggressive neuroblastomas have high p110alpha but low p110delta and p55alpha/p50alpha protein levels compared to low stage neuroblastomas

**DOI:** 10.1186/1750-2187-8-4

**Published:** 2013-04-18

**Authors:** Susanne Fransson, Per Kogner, Tommy Martinsson, Katarina Ejeskär

**Affiliations:** 1 Department of Medical and Clinical Genetics, Sahlgrenska Cancer Center, Institute of Biomedicine, Sahlgrenska Academy at University of Gothenburg, SE-405 30, Gothenburg, Sweden; 2 Department of Women’s and Children’s Health, Childhood Cancer Research Unit, Karolinska Institutet, SE-171 76, Stockholm, Sweden; 3 School of Life sciences, University of Skövde, SE-541 28, Skövde, Sweden; 4 Department of Medical and Clinical Genetics, University of Gothenburg, Sahlgrenska University Hospital, S-413 45, Gothenburg, Sweden

**Keywords:** Neuroblastoma, PI3K, Akt, Signaling, Phosphoinositide 3-kinase

## Abstract

**Background:**

The phosphoinositide 3-kinase (PI3K)/Akt pathway is involved in neuroblastoma development where Akt/PKB activation is associated with poor prognosis. PI3K activity subsequently activates Akt/PKB, and as mutations of PI3K are rare in neuroblastoma and high levels of PI3K subunit p110delta is associated with favorable disease with low p-Akt/PKB, the levels of other PI3K subunits could be important for Akt activation.

**Methods:**

Protein levels of Type IA PI3K catalytic and regulatory subunits were investigated together with levels of phosphorylated Akt/PKB and the PI3K negative regulator PTEN in primary neuroblastoma tumors. Relation between clinical markers and protein levels were evaluated through t-tests.

**Results:**

We found high levels of p-Akt/PKB correlating to aggressive disease and p-Akt/PKB (T308) showed inverse correlation to PTEN levels. The regulatory isomers p55alpha/p50alpha showed higher levels in favorable neuroblastoma as compared with aggressive neuroblastoma. The PI3K-subunit p110alpha was found mainly in advanced tumors while p110delta showed higher levels in favorable neuroblastoma.

**Conclusions:**

Activation of the PI3K/Akt pathway is seen in neuroblastoma tumors, however the contribution of the different PI3K isoforms is unknown. Here we show that p110alpha is preferentially expressed in aggressive neuroblastomas, with high p-Akt/PKB and p110delta is mainly detected in favorable neuroblastomas, with low p-Akt/PKB. This is an important finding as PI3K-specific inhibitors are suggested for enrollment in treatment of neuroblastoma patients.

## Background

Neuroblastoma is a pediatric cancer that stems from immature precursors of the sympathetic nervous system with tumors arising in sympathetic ganglia or adrenal gland. Neuroblastoma tumors display high clinical variability, ranging from mainly favorable stage 1 tumors to aggressive stage 4 tumors with high lethality. There are several markers that correlate to either the disease’s grade or outcome or both. Such markers are the expression of different Trk-receptors, degree of neural differentiation, or genetic aberrations such as 1p deletion, 11q deletion, gain of 17q, and amplification of the oncogene *MYCN*[1].

The phosphoinositide 3-kinase (PI3K)/Akt pathway participates in many biological processes such as proliferation, apoptosis, differentiation, metabolism and migration. The PI3K signaling cascade is initiated through activation of receptors with intrinsic tyrosine kinase activity, which leads to generation of the second messenger phosphatidylinositol (3,4,5)-triphosphate (PIP_3_), acting on downstream targets such as PI-dependent kinase (PDK1), integrin-linked kinase (ILK-1) or Akt/PKB. Type IA PI3K is a heterodimer composed of a regulatory subunit (p85α, p85β, p55α, p55γ or p50α) and a p110 catalytic subunit (p110α, p110β or p110δ). While p85β and p55γ is encoded by distinct genes, *PIK3R2* and *PIK3R3* respectively, p85α, p55α and p50α are different isoforms encoded by a single gene, *PIK3R1*.

Deregulation of the PI3K/Akt pathway is a recurrent feature in numerous human malignancies with a key role in cancer development, progression, and in resistance to chemotherapy. Over-activity is commonly caused by oncogenic activation of *PIK3CA*[2], over-stimulation caused by growth factors such as IGF-1, EGF or VEGF, or caused by loss of *PTEN* which is a negative regulator of Akt/PKB [3].

The contribution of PI3K/AKT in neuroblastoma carcinogenesis is not fully understood. Mutations in *PIK3CA* and *PTEN* are frequently reported in other malignancies but are rarely seen in neuroblastoma [4,5]. Although a few mutations have been reported in *PIK3CD*[6], *PIK3CD* shows lower expression levels in aggressive neuroblastoma tumors compared to tumors with more favorable biology [7]. Furthermore, activation of AKT is seen in neuroblastoma with a correlation to outcome [8]. In addition, several genes in the PI3K-pathway are differentially expressed in aggressive neuroblastoma as compared with favorable neuroblastoma [9]. PI3K signaling affects MYCN protein stability through inactivation of GSK3β. Furthermore, inhibition of PI3K destabilized MYCN and prevented tumor progression in a murine model of neuroblastoma [10].

PI3K inhibition is considered to be one of the most promising targeted therapies for cancer, therefore the understanding of the molecular pathology of the individual tumors will be essential in matching patients with PI3K inhibitors of differing selectivity profiles. In this study we have investigated the different catalytic and regulatory subunits of Type IA PI3K, the phosphatase PTEN as well as AKT-phosphorylation in primary neuroblastoma tumors and cell lines. The differences in PI3K-isoform expression pattern shown between tumor subgroups indicates that patients may benefit from targeted therapy with different isoform specific kinase inhibitors.

## Results

### Protein expression of the catalytic isoforms of the Type IA PI3-Kinase

**Figure 1:**
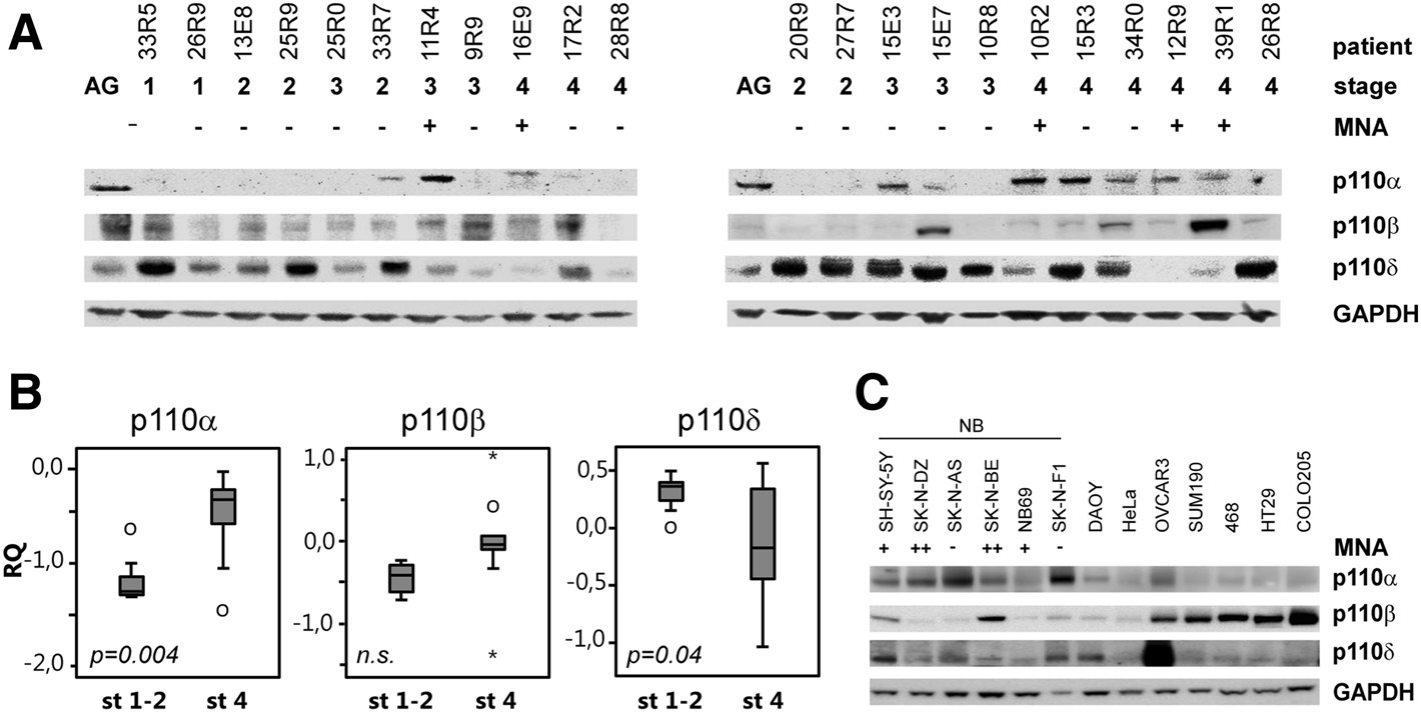
**Stage dependent expression of the different PI3K Type IA catalytic isoforms. A**) Protein levels of the different PI3K Type IA catalytical subunits were analyzed with western blot using two sets of primary neuroblastoma tumors. Abbreviations: AG, adrenal gland; MNA, MYCN amplification **B**) Relative quantification (RQ) of p110α, β and δ protein levels in primary tumors. Boxplot explanation; upper hinge of the box, 75^th^ percentile; lower hinge of the box, 25^th^ percentile; thick horizontal line within box, median. The whiskers are indicating range, open circles represent outliers while asterisks represent extremes. **C**) Western blot of cell lines with different origin show that cell lines of neuroblastic origin has high levels of p110α and low levels of p110β compared to non neuroblastic cell lines. Grading of MNA; no gain or amplification (−), gain (+), amplified (++).

To explore the different isoforms of the type IA PI3-kinase (p110α, p110β and p110δ), we performed western blot on lysates from 22 primary neuroblastoma tumors and normal adrenal gland as well as six neuroblastoma cell lines and seven cancer cell lines of different origin (medulloblastoma, cervix, ovary, breast and colon). All catalytic isoforms were detectable in adrenal gland and also at variable degree in NB tumors (Figure 1A). p110α was detected in 14% of stage 1–2 (1/7), 50% of stage 3 (3/6) and 78% of stage 4 (7/9). Protein levels were significantly higher in stage 4 neuroblastomas as compared with stage 1–2 (p = 0.004) (Figure 1B). Protein levels were also higher in *MYCN* amplified tumors compared to non-amplified tumors. (p = 0.001) (Additional file 1). High levels of p110α were also detected in neuroblastoma cell lines in general, compared to non-neuroblastoma cell lines. In contrast to primary tumors there was a trend of higher p110α levels in non-amplified neuroblastoma cell lines (SK-N-AS and SK-N-F1) compared to cell lines with 2p-gain (SH-SY-5Y and NB69) or *MYCN* amplification (SK-N-DZ and SK-N-BE) (Figure 1C). p110β was detected in 71% of stage 1–2 (5/7), 66% of stage 3 (4/6) and 89% of stage 4 (8/9) (Figure 1A). No major difference in protein levels of p110β levels were detected when comparing stage 4 and stages 1–2 neuroblastomas (Figure 1B), although higher levels were seen in *MYCN* amplified tumors compared to non-amplified tumors (p < 0.05) (Additional file 1). Cell lines originating from colon, breast, and ovaries had high levels of p110β compared to NB cell lines (p < 0.05) (Figure 1C). The PI3K Type IA catalytic subunit p110δ was detected in 100% of stage 1–2 (7/7), 86% of stage 3 (6/7) and 67% of stage 4 (6/9) (Figure 1A) with overall protein levels of p110δ significantly lower in stage 4 as compared with stage 1–2 neuroblastoma (p = 0.04) (Figure 1B). No significant difference was seen when comparing *MYCN* amplified and non-amplified samples (Additional file 1).

No significant difference was detected in correlation between age at diagnosis and protein levels of either PI3K Type IA catalytic subunit (Additional file 1).

### Protein expression of the regulatory isoforms of the Type IA PI3-Kinase

**Figure 2:**
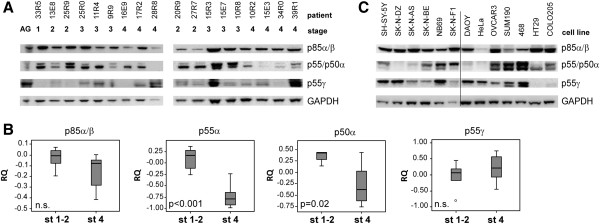
**Stage dependent expression of the PI3K Type IA regulatory isoforms. A**) Protein levels of the regulatory subunits of Type IA PI3K: p85α/β, p55α, p50α and p55γ were analyzed with western blot using two sets of primary neuroblastoma tumors. Abbreviations: AG, adrenal gland; **B**) Relative quantification (RQ) of p85α/β, p55α, p50α and p55γ protein levels using Gapdh as endogenous control. Boxplot explanation; upper hinge of the box, 75^th^ percentile; lower hinge of the box, 25^th^ percentile; thick horizontal line within box, median. The whiskers are indicating range, open circles represent outliers while asterisks represent extremes. **C**) Protein levels of regulatory subunits in neuroblastoma cell lines (to the left), and cell lines of other origins (to the right).

The protein levels of the different isoforms of the type IA PI3-kinase regulatory subunits (p85α/β, p55α, p50α and p55γ), were evaluated in primary NB tumors and cell lines. All isoforms were detectable in adrenal gland and to various extents in neuroblastoma tumors (Figure 2A). Quantification of p85α/β levels showed no statistical difference comparing stage 1–2 and stage 4 (Figure 2B). Whereas the slightly lower levels seen in *MYCN*-amplified tumors compared to non-amplified was below statistical threshold (p < 0.05) (Additional file 1). As judged by western blot, p85α/β were expressed at detectable levels in all cell lines (Figure 2C). The two shorter proteins isoforms p55α and p50α encoded by *PIK3R1* were detected at significantly lower levels in stage 4 compared to stage 1–2 neuroblastomas, p < 0.001 and p < 0.05 respectively (Figure 2B). p55α were detected in 5/6 (83%) and p50α in 4/6 (67%) of neuroblastoma cell lines (Figure 2C). The PI3K type IA subunit p55γ were detected in all cell lines except those of colorectal origin (Figure 2C) whereas a highly irregular pattern of protein expression was seen in primary tumors where protein levels ranged from undetectable to highly expressed (Figure 2A). No statistical difference in p55γ levels could be deducted when comparing stage 1–2 and stage 4 (Figure 2B), although a slightly higher level were seen in *MYCN* amplified compared to non-amplified tumors (p < 0.05) (Additional file 1).

No significant difference was detected in correlation between age at diagnosis and protein levels of either PI3K Type IA regulatory subunit (Additional file 1).

### Phosphorylation of Akt and correlation to PTEN levels

**Figure 3:**
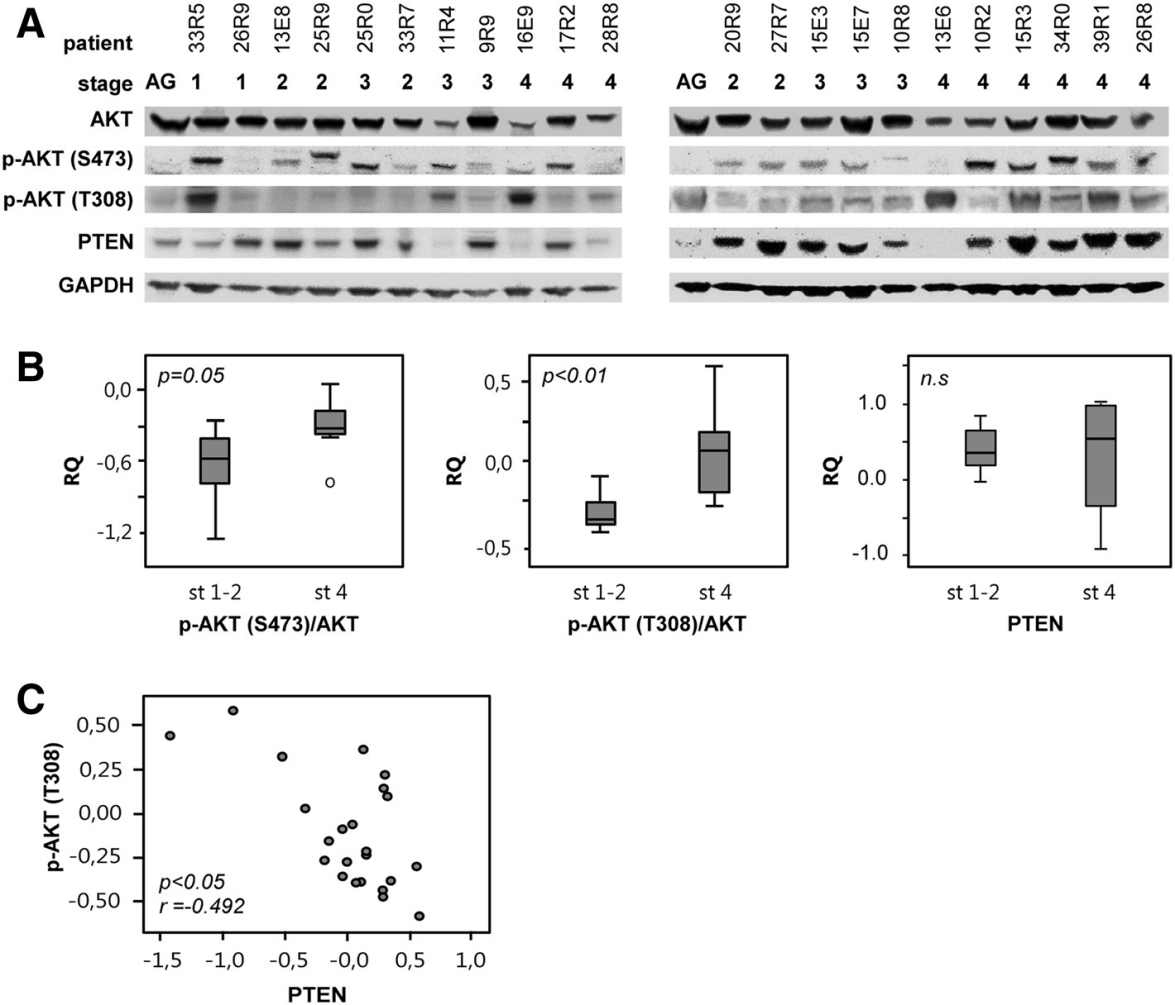
**Phosphorylated Akt and correlation to PTEN. A**) Western blot of primary neuroblastoma tumors showing levels of p-Akt/PKB (S473), p-Akt/PKB (T308), Akt/PKB and PTEN. AG = adrenal gland. **B**) Levels of p-Akt/PKB (T308), p-Akt/PKB (S473) and PTEN in stage 1–2 (st 1–2) and stage 4 (st 4) neuroblastoma tumors. Relative quantification (RQ) was calculated using GAPDH as endogenous control. **C**) Correlation plot using Spearman Rho showing that levels of phosphorylated Akt/PKB (T308) are inversely correlated with protein levels of PTEN.

Phosphorylation of Akt/PKB at serine 473 (S473) and threonine 308 (T308) were investigated by western blot in primary neuroblastoma tumors and analyzed in relation to protein levels of PTEN (Figure 3A). Levels of phosphorylated Akt/PKB (S473) (p = 0.05) and p-Akt/PKB (T308) (p < 0.01) in stage 4 neuroblastoma were significantly higher compared to stage 1–2 tumors (Figure 3B). Also, phosphorylation of Akt/PKB at T308 was inversely correlated with levels of PTEN (p = 0.02, correlation coefficient; -0.492, Spearmans Rho) (Figure 3C) while PTEN levels showed no significant difference when comparing stage 1–2 with stage 4 (Figure 3B) or comparing *MYCN* amplified with non-amplified.

## Discussion

The PI3K/Akt pathway is central for numerous cellular functions and it is frequently deregulated in human cancers. This pathway is also suggested to be an important player in neuroblastoma development where high mRNA expression of the PI3K catalytic isoform *PIK3CD* is associated with favorable disease [7], while activation of Akt/PKB is associated with poor prognosis [8]. These results look contradictory and warrant further evaluation of the different isoforms of PI3K in neuroblastoma, which was foreseen at the start of this study.

The tumor suppressor PTEN displays decreased activity in various cancers [3,11] and has also been implicated in neuroblastoma even though mutations in *PTEN* are rare in this tumor [5]. One report shows PTEN levels to be decreased in undifferentiated neuroblastomas [12] which could be due to localization of *PTEN* to the chromosome region 10q, commonly deleted in a subset of neuroblastomas. We noted no significant difference in the PTEN protein levels when comparing different clinical features of aggressive disease in our material (Figure 3B). However, we did detect a higher degree of phosphorylation of Akt/PKB at Ser473 and Thr308 in aggressive neuroblastoma and a statistically significant correlation between high levels of p-Akt/PKB (T308) and low protein levels of PTEN (Figure 3C), indicating a direct effect of PTEN levels and Akt/PKB (T308) phosphorylation.

PTEN activity can be modulated by the p85 regulatory subunit of the Type IA PI3K [13], which also enhances the phosphatase activity of PTEN [14]. Consequently, lower levels of p85 could lead to decreased PTEN activity and hence, increase Akt/PKB phosphorylation. Decreased mRNA-expression of *PIK3R1* has previously been detected in aggressive neuroblastoma [9], although no significant difference in p85 protein levels were seen comparing stage 1–2 and stage 4 tumors (Figure 2B) along with only a modest decrease in *MYCN* amplified tumors (Additional file 1). This indicates that increased Akt/PKB phosphorylation seen in stage 4 tumors is caused by other means than p85-modulated PTEN activity.

It is noted that the two shorter *PIK3R1* encoded isoforms p55α and p50α are both detected at statistically significant lower levels in stage 4 tumors compared to stage 1–2. It has been shown that the regulatory subunits have separate functions and that changes in expression can affect PI3K/Akt signaling [15,16]. One report suggests that p55α and p50α but not p85α are under transcriptional control of STAT3 [15], and a view of *STAT3* expression in the publically available R2 data base containing expression data from a set of 87 neuroblastoma tumors [17], show that STAT3 is significantly lower expressed in stage 4 tumors compared to stage 1 tumors (Additional file 2). However, to determine any causal relationship between p55α/p50α expression and STAT3 levels in a neuroblastoma context requires further investigation.

Oncogenic mutations of *PIK3CA,* encoding p110α, are common in various malignancies [2] but rare in neuroblastoma tumors [4], and there has been no reports of a difference in *PIK3CA*-mRNA expression between different stages of neuroblastoma. In a previous study no difference in *PIK3CA*-RNA-levels between different stages was found in this neuroblastoma material (Fransson et al. 2013). However, at the protein level we could detect significantly higher levels of p110α in aggressive tumors compared to stage 1–2 (Figure 1B), and also in MYCN amplified tumors compared to non-amplified. This indicates specific factors associated with aggressive disease could affect p110α stability or translation. An indication of this being a feature of neuroblastoma was the fact that the p110α protein levels were higher in neuroblastoma cell lines compared to the other tested cell lines (Figure 1C).

The p110β isoform has also been implicated in various malignancies [18,19], but we could not detect any difference between stage 1–2 and stage 4 neuroblastoma p110β protein levels (Figure 1B), although slightly higher levels were detected in MYCN amplified tumors (Additional file 1). In contrast, both mRNA and protein levels from *PIK3CD*/p110δ are decreased in stage 4 neuroblastoma compared to stage 1–2 as described by us and others previously [7,20].

Gene-targeting studies in mice have recently uncovered non-redundant roles for specific PI3K isoforms under normal physiological circumstances [21] but it is likely that different isoforms could have compensatory signaling in pathological conditions. Recent studies have shown that the α- and δ-isoform are downstream of receptor tyrosine kinases whereas p110β is activated through G-protein-coupled receptor signaling [22]. The pattern of high levels of p110δ in stage 1–2 and high levels of p110α in stage 4 (Figure 1B) indicate complementary functions of the α- and δ-isoform in Akt/PKB-signaling in neuroblastoma. However, the level of phosphorylated Akt/PKB is high in aggressive tumors where the level of p110α is high, indicating the importance of the α-isoform in advanced neuroblastoma.

## Conclusions

While the molecular mechanisms underlying neuroblastoma are slowly being uncovered, neuroblastoma continues to be fatal in many cases. In this study we show that the PI3K catalytic subunits p110α and p110δ are found in a pattern where p110α is detected at higher levels in aggressive tumors whereas p110δ is preferentially expressed in low stage tumors. Higher levels of p-Akt/PKB are associated with aggressive disease and indicate higher degree of activation of the PI3K/Akt pathway in these patients. The pattern of p110alpha and p110delta indicate complementary functions in Akt/PKB activation depending on neuroblastoma stage. Evaluation of the expression of the different PI3K isoforms in each neuroblastoma patient could be crucial for the success of treatment with PI3K isoform specific inhibitors.

## Methods

### Material

Freshly frozen tumor samples from patients diagnosed with neuroblastoma were staged according to the International Neuroblastoma Staging System Criteria (INSS) and International Neuroblastoma Risk Group (INRG) (Table 1). These were mainly post-treatment neuroblastomas, clinical data according to Table 1. The collection of material and subsequent study of the tissue material has been approved by the Karolinska University Hospital Research Ethics Committee (approval 03-736 and 2009/1369).

**Table 1:** Clinical data

**Patient**	**INSS**	**INRG**	**Age at diagnosis (months)**	**Follow up or outcome change (months)**	**Outcome**	**1p loss**	**MNA**	**11q loss**	**Origin**
26R9	1	L	2	91	NED	neg	neg	neg	Adrenal gland
25R9	2	L	111	95	NED	neg	neg	neg	NA
20R9	2	L	3	113	NED	neg	neg	NA	NA
27R7	2	L	0	92	NED	neg	neg	neg	Intraspinal abdominal
33R7	2	L	3	92	NED	neg	neg	neg	NA
13E8	2	L	32	202	NED	neg	neg	neg	NA
15E7	3	L	0	0	DSC	neg	neg	neg	NA
15E3	3	L	12	195	NED	neg	neg	neg	NA
25R0	3	L	1	97	NED	neg	neg	neg	Thorax
13E6	3	L	48	12	DOD	pos	pos	pos	NA
11R4	3	L	15	27	DOD	pos	pos	neg	NA
10R8	3	L	80	59	DOD	neg	neg	pos	NA
9R9	3	L	24	21	DOD	pos	neg	pos	NA
10R2	4	M	15	12	DOD	pos	pos	neg	NA
15R3	4	M	28	9	DOD	pos	neg	pos	Adrenal gland
34R0	4	M	64	11	DOD	neg	neg	neg	Abdominal
17R2	4	M	80	45	DOD	neg	neg	pos	NA
26R8	4	M	21	18	DOD	pos	pos	NA	NA
28R8	4	M	29	26	DOD	neg	neg	pos	Adrenal gland
16E9	4	M	19	5	DOD	neg	pos	neg	NA
39R1	4	M	22	29	NED	pos	pos	neg	Abdominal

Abbreviations: *INSS*, International neuroblastoma staging system; *INRG*, International neuroblastoma risk group; *MNA*, MYCN amplification; *NA*, Information not available; *L*, Localized; *M*, Metastasized; *NED*, No evidence of disease; *DOD*, Dead of disease; *DSC*, Dead by surgical complications; *pos*, Positive; *neg*, Negative.

The neuroblastoma cell lines SK-N-AS, SK-N-BE(2), NB69, SH-SY-5Y, SK-N-F1, SK-N-DZ together with HeLa (cervical cancer) and colon cancer cell lines HT29 and COLO205 were obtained from ECACC while DAOY (medulloblastoma), OVCAR3 (ovary adenocarcinoma) and MDA-MB-468 (breast carcinoma) were obtained from ATCC. SUM190 was a kind gift from Dr. Shubbar, Gothenburg University, Sweden. Previous genomic profiling of neuroblastoma cell lines shows that SK-N-BE(2) and SK-N-DZ have *MYCN* amplification and that SH-SY-5Y and NB69 have 2p-gain, while SK-N-F1 and SK-N-AS is copy neutral [23]. The cell lines were cultured according to standard procedures in DMEM, supplemented with 10% fetal bovine serum and 1% penicillin and streptomycin, before protein extraction according standard protocols.

### Immunoblotting

Tumors were homogenized using Tissuelyzer (Qiagen) in RIPA lysis buffer supplemented with HALT™ Phosphatase and protease inhibitor cocktail (Pierce, Rockford, IL). A ready-made protein lysate for normal adrenal gland (20 pooled donors) was purchased from Clontech (Mountain View, CA).

SDS-PAGE and Western blot were carried out according to standard procedures using 30 μg of total protein lysate. Immunoblotting was performed with rabbit polyclonal antibodies against AKT1/2/3 (sc-8312) p-Akt (S473) (sc-7985-R), p-Akt (T308) (sc-16646-R), PTEN (sc-7974), GAPDH (sc-25778), and p110δ (sc-7176) obtained from Santa Cruz Biotechnology (Santa Cruz, CA), pan-p85 (#06-496) from Millipore (Billerica, MA) while antibodies against p55γ (#11889), p110α (#4249) and p110β (#3011) were obtained from Cell Signaling Technology. Quantification of proteins was performed with the ImageJ software [24]. GAPDH was used for normalization in calculation of relative expression. The logarithms of expression levels were calculated and the difference between groups was assessed by a two-tailed independent-samples *t*-test. Correlations were calculated using Pearson bivariate analysis using the SPSS version 18.0 software (SPSS, Inc.).

## Competing interests

The authors have no competing interest that may influence this paper.

## Authors’ contributions

SF and KE designed research, performed research, interpret data and wrote the paper. TM and PK provided biological and clinical information. All authors read and approved the manuscript.

## Supplementary Material

Additional file 1 **Protein levels based on MYCN amplification or age at diagnosis.** Boxplot explanation; upper hinge of the box, 75^th^ percentile; lower hinge of the box, 25^th^ percentile; thick horizontal line within box, median. The whiskers are indicating range, open circles represent outliers while asterisks represent extremes. Click here for file

Additional file 2 **mRNA expression levels of STAT3. STAT3 show higher mRNA expression in stage 1 tumors compared to stage 4 tumors.** Boxplot derived from the R2: microarray analysis and visualization platform (http://r2.amc.nl). Click here for file
