# Supplementary figures and images for: Aggressive neuroblastomas have high p110alpha but low p110delta and p55alpha/p50alpha protein levels compared to low stage neuroblastomas
Source: J Mol Signal. 2013 Apr 18;8:4. doi: 10.1186/1750-2187-8-4 (PMC3639884; doi:10.1186/1750-2187-8-4)

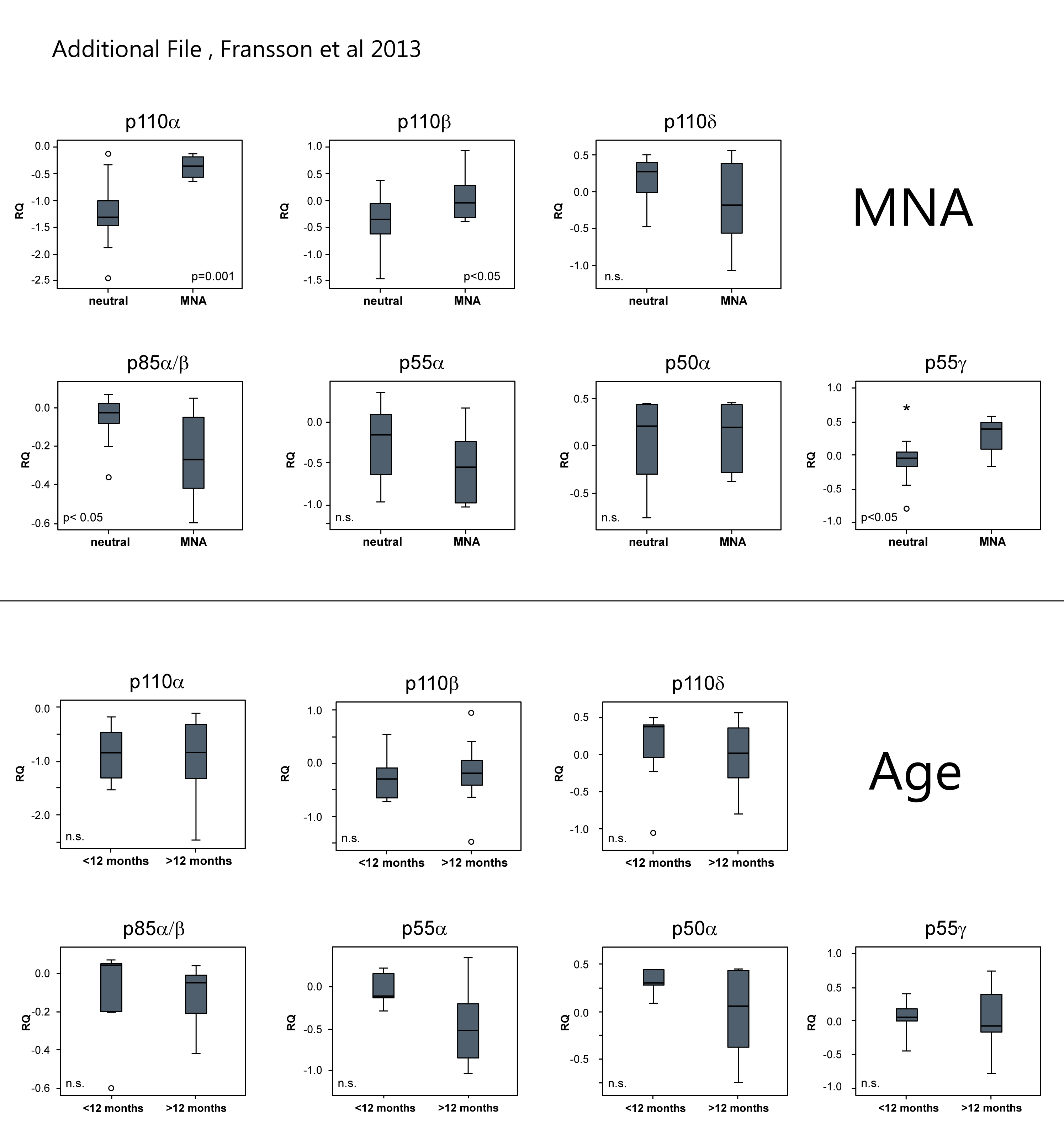

Supplement: Additional file 1 — Protein levels based on MYCN amplification or age at diagnosis. Boxplot explanation; upper hinge of the box, 75th percentile; lower hinge of the box, 25th percentile; thick horizontal line within box, median. The whiskers are indicating range, open circles represent outliers while asterisks represent extremes. [file 1750-2187-8-4-S1.tiff]

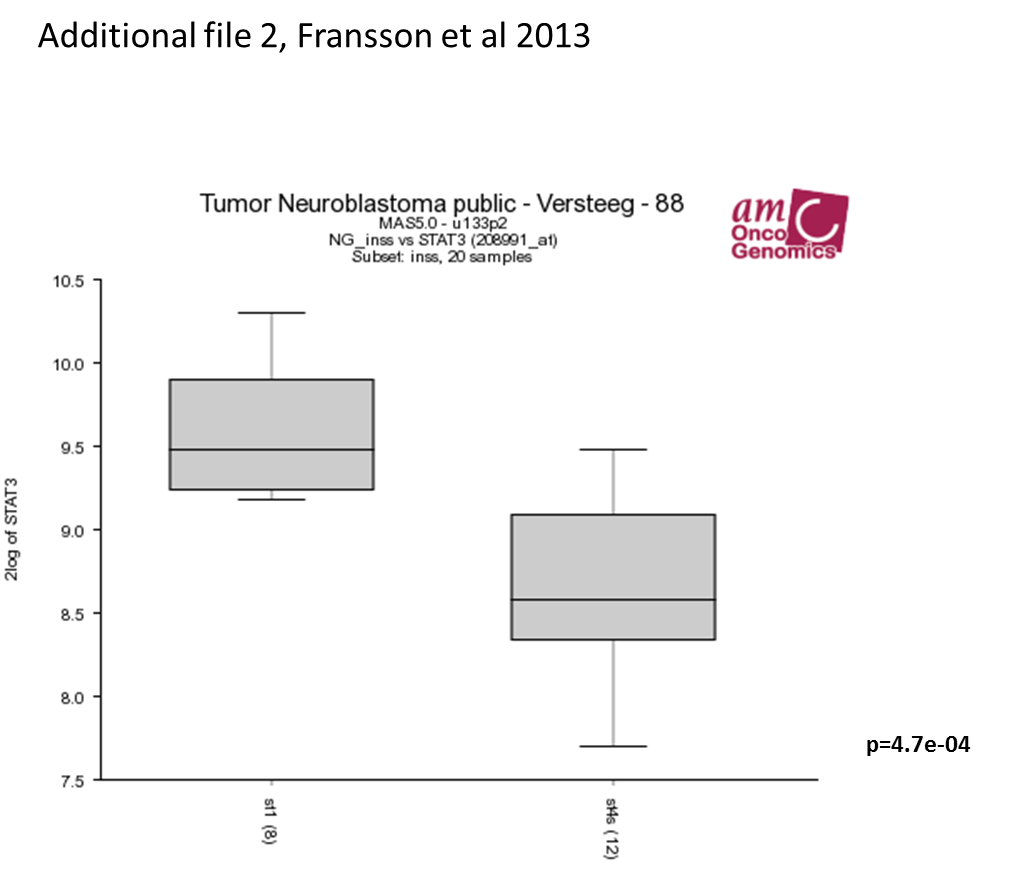

Supplement: Additional file 2 — mRNA expression levels of STAT3. STAT3 show higher mRNA expression in stage 1 tumors compared to stage 4 tumors. Boxplot derived from the R2: microarray analysis and visualization platform (http://r2.amc.nl). [file 1750-2187-8-4-S2.tiff]
